# Intraperitoneal ziv-aflibercept effectively manages refractory ascites in colorectal cancer patients

**DOI:** 10.18632/oncotarget.13543

**Published:** 2016-11-24

**Authors:** Chieh-Sheng Lu, Jen-Kou Lin, Wei-Shone Chen, Tzu-Chen Lin, Jeng-Kai Jiang, Shung-Haur Yang, Huann-Sheng Wang, Shih-Ching Chang, Yuan-Tzu Lan, Chun-Chi Lin, Hung-Hsin Lin, Hao-Wei Teng

**Affiliations:** ^1^ Department of Internal Medicine, Kaohsiung Armed Forces General Hospital, Kaohsiung, Taiwan; ^2^ Division of Hematology and Oncology, Department of Internal Medicine, Tri-Service General Hospital, National Defense Medical Center, Taipei, Taiwan; ^3^ Division of Medical Oncology, Department of Oncology, Taipei Veterans General Hospital, Taipei, Taiwan; ^4^ Institute of Clinical Medicine, National Yang-Ming University, Taipei, Taiwan; ^5^ School of Medicine, National Yang-Ming University, Taipei, Taiwan; ^6^ Division of Colon and Rectum Surgery, Department of Surgery, Taipei Veterans General Hospital & National Yang-Ming University, Taipei, Taiwan

**Keywords:** intraperitoneal injection, ziv-aflibercept, metastatic colorectal cancer, serum ascites albumin gradient

## Abstract

Ascites related to metastatic colorectal cancer (mCRC) reduces patient survival and quality of life, and systemic chemotherapy is largely ineffective for managing ascites. Here, we examined the clinical efficacy of intraperitoneal (IP) ziv-aflibercept for managing refractory ascites in 15 mCRC patients who did not respond to standard chemotherapy. Fifty or 100 mg of ziv-aflibercept in 100 mL of saline solution were infused through a pigtail catheter and retained for 24 h. When the ascites drainage volumes were subsequently monitored, 73.3% of patients showed an objective response (OR) to IP ziv-aflibercept treatment. Patients with low Eastern Cooperative Oncology Group (ECOG) performance status or with serum ascites albumin gradients (SAAG) less than 1.1 g/dL had better responses to treatment, and 4 patients with SAAG less than 1.1 g/dL showed rapid objective responses (rOR). These findings indicate that intraperitoneal ziv-aflibercept therapy may be a highly effective means of treating refractory ascites in mCRC patients, and that SAAG may be predictive of a rapid response to this treatment.

## INTRODUCTION

Approximately half of colorectal cancer (CRC) patients eventually develop metastatic colorectal cancer (mCRC) [1–3]. Advanced mCRC may cause peritoneal carcinomatosis and/or massive liver metastases, which in turn contribute to the formation of ascites due to the accumulation of fluid produced by tumor cells in the peritoneal cavity, the obstruction/compression of the portal veins, liver failure, or increased vascular permeability. These ascites often increase intra-abdominal pressure and are associated with abdominal dullness, bloating, early satiety, fatigue, and nausea in patients. As fluid continues to accumulate in the abdominal cavity, patient mobility and quality of life continue to decline, and ascites may decrease patient survival [4].

Traditionally, therapeutic paracentesis and diuretics have been used as palliative treatments for ascites resulting from mCRC. Unfortunately, patients often develop refractory ascites [5, 6]. Intraperitoneal chemotherapy (IPC), in which therapeutic drugs are injected into the peritoneal cavity, have been studied in the past as a potential treatment strategy. In 1955, Weisberger *et al*. found that intraperitoneal (IP) nitrogen mustard treatment slowed the growth of malignancy-related ascites in seven ovarian cancer patients [7, 8]. However, due to a lack of efficacy in the treatment of intra-abdominal tumors and considerable toxicity in early clinical studies, IPC was largely abandoned in 1980s [8, 9], although it is still used as an alternative treatment for stage III ovarian cancer patients after optimal debulking surgery [10]. In addition, hyperthermal IPC (HIPEC) has been used after cytoreductive surgery to treat mCRC; however, the use of IPC with conventional chemotherapy agents remains controversial.

Bevacizumab downregulates vascular endothelial growth factor (VEGF), the most easily-assessed target in mCRC, by binding to the VEGF-A ligand, thus inhibiting angiogenesis, microvascularity, and tumor proliferation [11–17]. VEGF is elevated in malignant ascites and contributes to their growth by increasing endothelial permeability [18]. IP bevacizumab administration is effective in treating ovarian cancer and has the palliative benefit of preventing the recurrence of ascites [19–21]. However, the use of bevacizumab for treating ascites in mCRC has not yet been examined in clinical trials.

Ziv-aflibercept, a recombinant protein that inhibits the VEGF pathway, binds to VEGF-A, VEGF-B, and placental growth factor (PlGF) [22]. In animal models, ziv-aflibercept has stronger anticancer effects than bevacizumab in CRC, suggesting that targeting both VEGF/PlGF is more beneficial than targeting either ligand alone [23]. In clinical practice, ziv-aflibercept is used as a second-line agent for treating mCRC. However, its effectiveness in treating ascites resulting from mCRC remains unknown. Here, we examined the effects of IPC with ziv-aflibercept on mCRC-related ascites in a clinical study at our institution.

## MATERIALS AND METHODS

### Study design

This retrospective study was conducted using population-based data from the Taipei Veterans General Hospital, Taipei, Taiwan, under the guidelines of the Helsinki Declaration. The study was approved by the Human Subjects Protection Offices (IRB) at the Taipei Veterans General Hospital (VGHIRB number: 2015-08-004CC). Since all identifying patient information was removed prior to the study, informed consent was not obtained.

Patients at Taipei Veterans General Hospital with confirmed pathologic colorectal adenocarcinoma with distant metastasis were enrolled between January 2014 and June 2016. All patients had failed to respond to standard bio-chemotherapy that included bevacizumab, cetuximab, irinotecan, 5-FU, and oxaliplatin, and suffered from colorectal ascites. Only patients who received IPC with ziv-aflibercept were enrolled in this study. Basic clinicopathological parameters, including age, gender, body weight, abdominal girth, amount of drained ascites, ascites analysis, tumor location, metastatic sites, serum cell counts, serum biochemistry examinations, and serum ascites albumin gradient (SAAG) were evaluated. SAAG was calculated by subtracting the albumin concentration of the ascites fluid from the albumin concentration of a serum specimen obtained on the same day [24].

During IP ziv-aflibercept therapy, a sonography-guided pigtail catheter was first inserted into abdominal cavity for ascites treatment. Paracentesis was then performed for two days to remove 1.5 to 2 liters (L) of ascites; IPC with ziv-aflibercept was performed on day 3. Fifty or 100 milligrams (mg) of ziv-aflibercept diluted in 100 milliliters (mL) of normal saline was instilled over 15 minutes through the pigtail catheter. Daily paracentesis of no more than 1.5 L per day began 24 hours later. Ziv-aflibercept doses were adjusted at the attending physician's discretion to avoid contraindications. Ascites drainage volumes were recorded daily.

### Evaluation of response

Treatment response was evaluated by assessing patient symptoms, the amount of ascites drained, abdominal girth, changes in body weight, and the timing of repeated paracentesis. Objective response rate (ORR) (Table 1) was defined by improved clinical features and revised refractory ascites according to the following criteria: (1) symptoms improved, (2) ascites amounts decreased, (3) abdominal girth decreased, and (4) no early ascites recurred [reappearance of grade 2 or 3 ascites (clinically detectable or more than 500 mL) within 4 weeks of initial mobilization] or a loss of more than 0.8 kilograms (kg) of bodyweight during the 4 days after initial IP treatment [5, 6]. Rapid objective response (rOR) was defined as a daily ascites tap volume of less than 0.5 L within 7 days of IPC. Complications or poor responses after IP ziv-aflibercept were considered failures of treatment.

**Table 1:** Definition of objective response of intraperitoneal ziv-aflibercept

1.	Clinical features improved:
	Symptoms improved, and
	Ascites decreased, and
	Abdominal girth decreased
2.	Refractory ascites revised:
	No reappearance of grade 2 or 3 ascites (clinically detectable or more than 500 milliliters) within 4 weeks of initial mobilization, or body weight loss of more than 0.8 kilogram (kg) over 4 days after initial intraperitoneal treatment.

### Statistical analysis

Patient characteristics analyzed included sex, gender, tumor location [25], liver metastasis (Mets), Eastern Cooperative Oncology Group (ECOG) performance status (PS), serum white blood cell counts (WBC), hemoglobin (Hb) levels, platelet counts (PLT), and SAAG. The distribution of baseline patient characteristics across OR was evaluated using Pearson's X^2^test for categorical variables. All *P* values were two-sided; *P* less than 0.05 was considered statistically significant. Data were analyzed using the Statistical Package for the Social Sciences (SPSS, IBM PASW Statistics 18, version 18.0.0, WinWrap Basic, copyright 1993–2007, Polar Engineering and Consulting).

## RESULTS

### Patient characteristics

Patient characteristics, and the distribution of those characteristics in patients with different therapeutic responses, are shown in Table 2. The median observation period (beginning on the date of IP ziv-aflibercept and ending on the final date of the study) in all patients was 12.0 weeks. Treated patients had a median age of 56.0 years (range 31–81); 5 patients were at least 70 years old at the time of treatment. Nine of the 15 patients included in this study were men and 6 were women. Four patients were diagnosed by imaging with cancer tumors initially located in the right colon. Thirteen of the 15 patients had liver metastasis. Twelve patients had an ECOG performance status (PS) of 2, and the remaining 3 patients had an ECOG PS of 3. Before treatment, 3 patients had leukocytosis (WBC>10000/mm^3^), 12 had anemia (Hb<12 g/dl), and 5 had thrombocytopenia (PLT<150 x10^3^/mm^3^). SAAG data was available for 10 patients, of which 4 had SAAG ≥ 1.1 g/dL. Nine patients received 50 mg of ziv-aflibercept during IP infusion, while the remaining 6 patients received 100 mg.

**Table 2:** The descriptive characteristics and distribution of patients according to objective response of treatment

Patients’ characteristics	No. of total patients	OR	Failure	*P*
No. of patients	15	11 (73.3%)	4 (26.7%)	
Age (y/o)				
Range	31-81			
Median	56.0			
Mean	59.5±13.7			
≧70	5	4	1	.680
<70	10	7	3	
Gender				
Male	9	6	3	.475
Female	6	5	1	
Tumor Site				
R-colon	4	3	1	.930
L-colon	11	8	3	
Liver Mets				
Y	13	9	4	.360
N	2	2	0	
ECOG PS				
2	12	11	1	.001
3	3	0	3	
WBC (/mm^3^)				
Range	2300-37200			
Median	5900.0			
Mean	8306.0±8447.8			
>10000	3	1	2	.080
≦10000	12	10	2	
Hb (g/dl)				
Range	6.9-13.8			
Median	10.8			
Mean	10.9±1.7			
≧12	3	2	1	.770
<12	12	9	3	
PLT (x10^3^/mm^3^)				
Range	73-449			
Median	203.0			
Mean	212.8±111.5			
≧150	10	8	2	.409
<150	5	3	2	
SAAG (g/dl)*				
Range	0.48-1.96			
Median	1.035			
Mean	1.084±0.468			
≧1.1	4	1	3	.011
<1.1	6	6	0	
Dosage of ziv-aflibercept				
50mg	9	8	1	.095
100mg	6	3	3	
Follow-up (weeks)				
Range	0.0-55.0			
Median	12.0			
Mean	18.9±17.9			

* Missing data in 5 patients.

Abbreviations: ECOG: Eastern Cooperative Oncology Group performance status; Hb: hemoglobin; L-colon: left side colon cancer; Mets: metastasis; N: no; No.: number; OR: objective response; *P*: probability value; PLT: platelet; R-colon: right side colon cancer; SAAG: serum ascites albumin gradient; WBC: white blood cell; Y: yes; y/o: years old.

### Analysis of objective response

**Figure 1:**
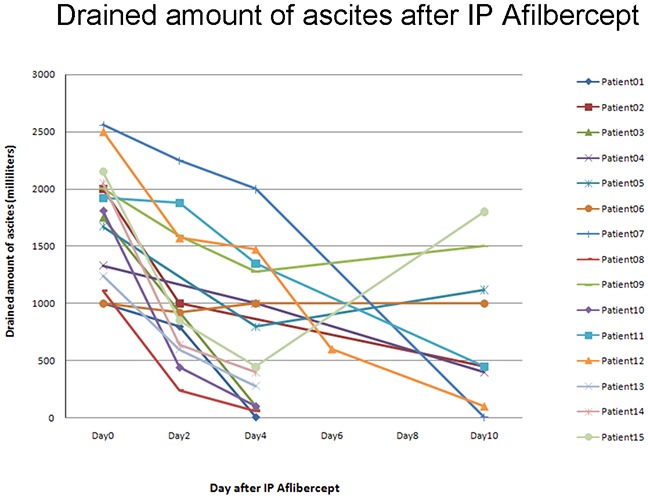
The objective response rate (ORR) for the 15 patients enrolled in the study was 73.3% Refractory ascites persisted in patients 05, 06, 09 and 15. In addition, 6 patients (40.0%) showed rapidly objective responses (rOR) along with a dramatic reduction in ascites.

Symptoms improved and body weight and abdominal girth decreased in all patients. In addition, ascites decreased in all patients except for patient 06. More than 500 mL of ascites reappeared within 4 weeks in 3 patients, and only 11 patients (73.3%) met the definition of objective response (OR) (Table 2). Pearson's X^2^ test for categorical variables revealed correlations between ECOG PS and OR (*ρ* = -0.829, *P* = 0.001) and between SAAG < 1.1 g/dL and OR (*ρ* = 0.802, *P* = 0.011), indicating that IPC was more effective in controlling ascites in patients with low ECOG PS or SAAG < 1.1 g/dL. In addition, there were trends towards correlations between WBC>10000/mm^3^ and OR (*ρ* = -0.452, *P* = 0.080) and been dosage 50mg/100mg and OR (*ρ* = -0.431, *P* = 0.095), although they did not reach statistical significance. These results suggest that patients with WBC ≤ 10000/mm^3^ or who receive ziv-aflibercept doses of 50mg may respond to ascites control IPC treatment. Therapeutic efficacy as indicated by decreases in drained ascites amounts over time are shown in Figure 1. Rapid OR (rOR) occurred after IPC in 6 patients (40.0%) (Table 3). Four patients (patients 08, 10, 13, and 14) with SAAG < 1.1 g/dL showed rOR (*P* = 0.035), and lower SAAG values were associated with rOR. No other parameters, including ECOG PS, ziv-aflibercept dosage, and WBC before treatment, were correlated with rOR. Low SAAG values may therefore accurately predict rOR in patients with refractory ascites after IP ziv-aflibercept treatment.

**Table 3:** Subgroup analysis relation to rapidly objective response

Patient	ECOG PS	DOSAGE(mg)	WBC (/mm^3^)	SAAG(g/dl)	rOR	OR
01	2	50	10600	--	O	O
02	2	50	9200	--	X	O
03	2	50	2500	--	O	O
04	2	50	4800	--	X	O
05	3	100	10400	--	X	X
06	3	100	37200	1.72	X	X
07	2	100	4760	1.05	X	O
08	2	50	7870	**0.67**	**O**	O
09	2	100	9400	1.12	X	X
10	2	100	6380	**0.48**	**O**	O
11	2	50	4600	1.02	X	O
12	2	100	3100	1.96	X	O
13	2	50	5580	**0.72**	**O**	O
14	2	50	5900	**0.8**	**O**	O
15	3	50	2300	1.3	X	X

Abbreviations: O: yes; X: no; ECOG: Eastern Cooperative Oncology Group performance status; OR: objective response; rOR: rapidly objective response; SAAG: serum ascites albumin gradient; WBC: white blood cell

### Adverse events

None of the patients died during the follow-up period; median overall survival (OS) therefore could not be evaluated. None of the patients experienced drug-related adverse events, including bleeding, perforation, thrombosis, hypertension, proteinuria, and malaise.

## DISCUSSION

Here, we found that IPC with ziv-aflibercept was effective in treating ascites caused by mCRC without causing adverse events. Furthermore, our results indicate that SAAG might serve as a good predictive marker of rapid therapeutic response to IPC treatment.

Because the use of IPC with ziv-aflibercept in mCRC has not yet been evaluated in clinical trials, we compared our results with to those obtained using IPC with bevacizumab in ovarian cancer patients. The impressive response rates and minimal adverse events seen here in mCRC patients after IPC with ziv-aflibercept are similar to those observed previously in ovarian cancer patients who received IPC with bevacizumab to treat ascites [11, 19–21, 26]. Chad *et al*. reported that ascites and bilateral lower limb edema dramatically improved 4 days after IPC with bevacizumab in ovarian cancer patients. Similarly, 6 patients (40.0%) in this study showed rOR within 7 days of IPC. Moreover, ascites was substantially reduced in 73.3% of mCRC patients after one month of IPC. This is consistent with a previous study in which 58 patients with ovarian epithelial cancer accompanied by malignant ascites received IP administration of either cisplatin alone (control) or together with 300 mg of bevacizumab every 2 weeks for 6 weeks [21]. Treatment with both cisplatin and bevacizumab decreased VEGF levels in ascites in compared to controls. Furthermore, ORR and quality of life were higher in the bevacizumab-treated group than in the control group (90.32 vs. 59.26%, *P* < 0.05, and 93.55 vs. 48.15%, respectively; both *P* < 0.05) [21].

There are several possible explanations for the high response rates and minimal adverse events observed here. First, the peritoneal-plasma barrier allows for the administration of high IPC drug concentrations due to its ability to reduce systemic absorption and associated toxicity [27]. These low levels of diffusion and tissue penetration may partially explain the minimal adverse events observed here after treatment [28]. Kraft *et al*. first suggested that IPC with anti-VEGF antibodies might be effective for treating ascites, which have much higher VEGF levels than matched sera [29]. In that study, local release of VEGF, a cytokine originally called vascular permeability factor, by ascites in the peritoneal cavity might have contributed to hyperpermeability in the microvasculature of tumors and the peritoneum. Directly targeting malignancy-related peritoneal ascites, in which VEGF concentrations are highest, may therefore improve therapies for patients receiving primarily palliative treatment [19]. Ziv-aflibercept, a recombinant protein consisting of the fragment crystallizable (Fc) region of human immunoglobulin G1 (IgG1) fused to segments of human VEGF receptors 1 and 2, has a high affinity for VEGF, and thus inhibits angiogenesis induced by activation of VEGF receptors [22]. In addition, ziv-aflibercept, which binds VEGF-A, VEGF-B, and PlGF, has higher affinity and efficacy than bevacizumab, which binds only VEGF-A [23, 30]. A recent study treated mCRC patients from the VELOUR trial with ziv-aflibercept as a second-line therapy [31]. In mice that received mCRC xenografts and intraperitoneal treatment with VEGF-Trap, a soluble decoy VEGF receptor, ascites was eliminated and tumor burdens decreased by 56% compared to controls [32]. In Taiwan, national insurance pays for treatment with bevacizumab but not ziv-aflibercept. Thus, the patients receiving ziv-aflibercept therapy in this study were under palliative treatment plans or paid for the treatment themselves after other front-line therapies failed.

Perhaps the most important result of the current study was that IPC with ziv-aflibercept resulted in a 73.3% ORR, as indicated by reductions in refractory ascites, among the 15 included mCRC patients. No drug-related adverse bleeding, thrombosis, hypertension, proteinuria, or malaise events were disclosed. This ORR of 73.3% was greater than the 62.5% ORR observed in a phase II study of systemic intravenous ziv-aflibercept in advanced ovarian cancer patients who had symptomatic malignant ascites [33]; toxicity was also lower in our study. In addition, 6 patients showed rOR (40.0%) and dramatic decreases in ascites, and low SAAG values in these patients predicted positive responses to this IP treatment [34].

The dosage of ziv-aflibercept used in IPC therapy also affects the efficacy of this treatment. Three of the 6 patients (50.0%) who received 100 mg of ziv-aflibercept by IP instillation did not respond to treatment during the follow-up period. In contrast, 8 of the 9 patients (88.9%) who received 50 mg of ziv-aflibercept by IP instillation showed objective responses. High IP does of ziv-aflibercept therefore seem to be less beneficial. Future studies should investigate the optimal dosage and dose-related toxicity of IP ziv-aflibercept in mCRC.

Some important biases in this single-institution observational study of a small patient cohort should be considered when interpreting the results. First, we did not analyze ascites cytology in all patients. Emoto *et al*. found that periodic evaluation of peritoneal lavage fluid cytology is helpful when evaluating the efficacy of IPC and in predicting outcomes in gastric cancer patients with peritoneal dissemination [35]. These findings may also apply to patients with other types of gastrointestinal cancer involving malignancy-related ascites. Our data also indicate that low SAAG values may predict successful reduction of ascites in response to IP ziv-aflibercept, and it is possible that low SAAG values may also predict reduced peritoneal carcinomatosis in the CRC patients. However, additional large-scale prospective studies of this IPC treatment including detailed cytological examination of ascites should be conducted to confirm these associations with SAAG values. Secondly, we did not evaluate molecular profiles in advanced analyses in advanced cancer patients for whom front-line treatments were unsuccessful. Although molecular profiling can be clinically useful for evaluating responses to treatment in colorectal cancer, it is still not routinely included in cancer management strategies. In addition, collecting and preserving ascites specimens is difficult. Due to the lack of molecular information in this study, we examined only clinicopathological characteristics and therapeutic factors in our analysis. Integrating various molecular characteristics may improve the accuracy of outcome predictions for IPC treatment. Quality of life has not typically been evaluated in past studies [36], perhaps due to the many confounding factors involved, such as unidentified comorbidities and the aggressiveness of first-line treatments. We therefore did not investigate the effects of our IPC treatment on quality of life in this study. Finally, a very small number of patients was included in this study, and the results may therefore have limited applicability in larger patient populations.

Nevertheless, our results suggest that IPC treatment with ziv-aflibercept may be a valuable alternative strategy for reducing refractory ascites in mCRC patients. In addition, pre-treatment SAAG values may predict the efficacy of IP ziv-aflibercept. We thus recommend the use of IP ziv-aflibercept for the management of refractory ascites in mCRC patients.
